# Dental pain associated with untreated dental caries and sociodemographic factors in 5-year-old children

**DOI:** 10.4317/jced.57827

**Published:** 2021-06-01

**Authors:** Suyene-de Oliveira Paredes, Renato-Ferreira da Nóbrega, Thays-da Silveira Soares, Maria-Eliza-Dantas Bezerra, Mauro-Henrique-Nogueira-Guimarães de Abreu, Franklin-Delano-Soares Forte

**Affiliations:** 1 Post-graduation Program in Dentistry. Federal University of Paraíba, João Pessoa, Paraíba, Brazil. University City, 58.051-900, João Pessoa, Paraíba, Brazil; 2 Dentistry Course. University Center of Patos, 58704-000, Patos, Paraíba, Brazil; 3 Federal University of Minas Gerais, Belo Horizonte, Minas Gerais, Brazil

## Abstract

**Background:**

The aim of this study was to assess the prevalence of dental pain and to examine its association with untreated dental caries in 5-year-old children.

**Material and Methods:**

This was a cross-sectional study of 261 5-year-old children in Northeast Brazil. Parents answered questions about their socioeconomic conditions and their children’s toothache. Trained dentists assessed dental caries using the decayed, missing and filled teeth (dmf-t) index. Binary logistic regression models were used to estimate the unadjusted and adjusted odds ratios (ORs) and the confidence intervals (95% CIs) for the association of dental pain with covariates. The significance level was set at five percent.

**Results:**

The prevalence of dental pain was 28.7%, and 48.2% of children had untreated dental caries. Dental pain was associated with untreated dental caries (OR=5.7; 95% CI: 3.1-10.53; *p*<0.001) and living with one parent or other family members (OR=2.3; 95% CI: 1.2-4.4; *p*=0.008). Sociodemographic factors were not associated with dental pain.

**Conclusions:**

The prevalence of dental pain in preschool children is high, and this condition is associated with both untreated dental caries and living with one parent or other family members.

** Key words:**Toothache, socioeconomic factors, dental caries.

## Introduction

Dental pain is a relatively common condition in children and impacts the lives of not only children but also their families (1,2). Although dentistry has advanced in recent years, reports of toothache are still very frequent, and toothaches lead to suffering, illness, discomfort while eating, discomfort while brushing teeth, sleep disturbance, school absenteeism, and impaired social interactions (3-5). Therefore, it is important to assess dental pain due to its impact on children and their family’s lives (6-8).

In addition to its impact on children and their family, dental pain is a common complaint in oral health services (6-9). The prevalence of toothache in 5-year-old children ranges from 7- 25% (10-13). At the national level, the prevalence of dental pain in preschoolers varies between 7.2% and 22% (3,4,7,9,14,15).

Identifying pain in children is challenging because children of preschool age have difficulties in expressing their feelings clearly due to their restricted communication skills. Thus, it is possible for a child to experience toothache at a certain moment and not seek the appropriate treatment, leading to the worsening of this condition (3,6,7).

Some factors have been shown to be associated with dental pain in children aged up to 5 years, such as sociodemographic factors, the Human Development Index (HDI), family income, number of residents per room, parent’s educational level, not having a father or mother, age, ethnicity, use of dental services, and clinical factors such as a history of dental caries, oral conditions and the physiological mobility of primary teeth in the exfoliation process (1,3,4,6,9,14).

It has been reported that dental caries are a major cause of toothache (3,11,14,16). A global study found that 573 million children have untreated caries in primary teeth (11).

An understanding of how toothache manifests in children aged up to 5 years is important for the implementation of strategies to combat this issue and for the establishment of health promotion programs that take into account cultural, economic, and intersectoral aspects. The actions and activities related to primary oral health care are reflected by the low cost of treatment (16,17).

Most existing studies regarding the relationship between dental pain, sociodemographic factors, and untreated caries in 5-year-old children were conducted in large urban centers. There are no studies in Brazilian cities with small population sizes, which have different characteristics than large urban centers. The aim of this study was to assess the prevalence of dental pain and to examine its association with untreated dental caries in 5-year-old Brazilian children.

## Material and Methods

This study is reported according to the STROBE checklist. The study was accepted by the local Human Research Ethics Committee under two protocols (No. 3,127,183 and No. 3,131,857) in compliance with the Brazilian National Health Council Resolution 466/2012 and the Helsinki Declaration. Informed consent for a clinical assessment was signed by the children’s parents or guardians and contained information about the purpose of the study.

-Sample characteristics and study design

This was a cross-sectional, censitary, descriptive, and analytical study conducted in two cities in northeastern Brazil: Santa Luzia, which has a population of 11,788 inhabitants, HDI=0.682, and Gini index=0.533; and Paulista, with 4,830 inhabitants, HDI=0.625, and Gini index=0.496.

Cross-sectional and census study was carried out involving male and female children aged five years old enrolled at public educational institutions in rural and urban area of Santa Luzia (N=150) and Paulista (N=171).

-Eligibility criteria

The participants were selected from each of the educational institutions based on attendance lists provided by teachers. Children were 5 years old who did not have a systemic disease (according to the parents and/or teachers’ reports). Clinical examinations were attempted three times. Children were excluded if they (or their parents) did not speak Portuguese, had a health problem, did not allow the clinical examination, or were not authorized by their guardian to participate.

-Training

The training was divided into two stages: the first stage was theoretical, in which the World Health Organization (WHO) criteria and codes for digital dental photo analysis (18) were discussed; the second stage was clinical, in which 20 children were examined and reexamined (intraexaminer agreement was excellent, k=0.83-0.98). The two training stages lasted for a total of 8 hours and were coordinated by a specialist.

-Pilot study

A pilot study was carried out to analyze the study methodology. Twenty children participated in this pilot study. Children participating in this stage were not included in the final study population. As there were no problems with the application questionnaires addressing sociodemographic data as well as the Brazilian version of the Early Childhood Oral Health Impact Scale (B-ECOHIS) (19), including history of dental pain, there was no need for changes in the study methodology.

-Data collection

Data collection took place in educational institutions in the two cities. Initially, contact was made with the persons in charge of the institutions to explain the purpose of the study. Then, a free and informed consent form and the study questionnaire were sent to the parents.

The questionnaire was addressed to the parents or guardians of the children and assessed the following sociodemographic characteristics: independent variables (dichotomized): sex (male/female), people living with the child (father, mother, both, or someone else), housing status (own vs rent), median number of rooms (up to 4/more than 4), family income (below minimum wage/more than minimum wage), mother’s age (below 35 years old/over 35 years old), mother’s education (less than 8 years of study/more than 8 years of study), father’s education (less than 8 years of study/more than 8 years of study), mother working, mother’s marital status (single/divorced/married/other), and number of siblings (1-2/more than 2).

The dependent variable “toothache” was investigated using the following question: “has your child ever had pain in the teeth?” from the B-ECOHIS. The answer was dichotomous (absent/present).

The clinical examination was performed after tooth brushing under natural light with the aid of gauze, a mouth mirror (PRISMA®, São Paulo, SP, Brazil), and a WHO probe (Millenium®). The instruments were properly sterilized. The examination was performed with the participants sitting in a common chair and facing the examiner, who was wearing protective goggles, gloves, a hat, and a mask. To evaluate dental caries, the WHO caries index and treatment needs were used. For statistical analyses, the presence or absence of untreated dental caries (decayed component of the dmf-t index that corresponds to decayed, missing and filled teeth) was considered the independent variable.

-Statistical analysis

All analyses were conducted using the Statistical Program for Social Sciences software version 20.0 (SPSS for Windows, SPSS, Inc., Chicago, IL, USA). Binary logistic regression models were used to estimate the unadjusted and adjusted odds ratios (ORs) with confidence intervals (95% CIs) to determine the association between dental pain and the covariates. Each covariate was included separately in the regression model, and the unadjusted OR was estimated. Covariates with *p-value*s less than 0.25 were included in the final conditional binary logistic model, where only variables that had *p-value*s less than 0.05 were maintained. The Hosmer- Lemeshow test was used to evaluate the goodness-of-fit of the final model.

## Results

From the whole population of children in both towns, 120 children were included in Santa Luzia (response rate = 80.0%) and 141 children in Paulista (response rate = 82.5%). Losses occurred due to consecutive children’ absence from educational institutions after three attempts for dental examination, refusals to participate, and lack of cooperation during the exam.

The prevalence of dental pain was 28.7%. Table 1,  Table 1 cont. shows the characteristics of the analyzed variables. The presence of untreated dental caries increased the odds of dental pain (OR=5.7; 95% CI: 3.1-10.5). Children who did not live with their parents were more likely to have toothache (OR=2.3; 95% CI: 1.2-4.4) (Table 1,  Table 1 cont.). The Hosmer-Lemeshow test indicate that model was a good fit (*p*=0.782). Despite this study was a census, a posteriori power calculation was carried out for untreated dental caries and dental pain considering 95% confidence interval, resulting in a power value higher than 99.0%.

**Table 1:**
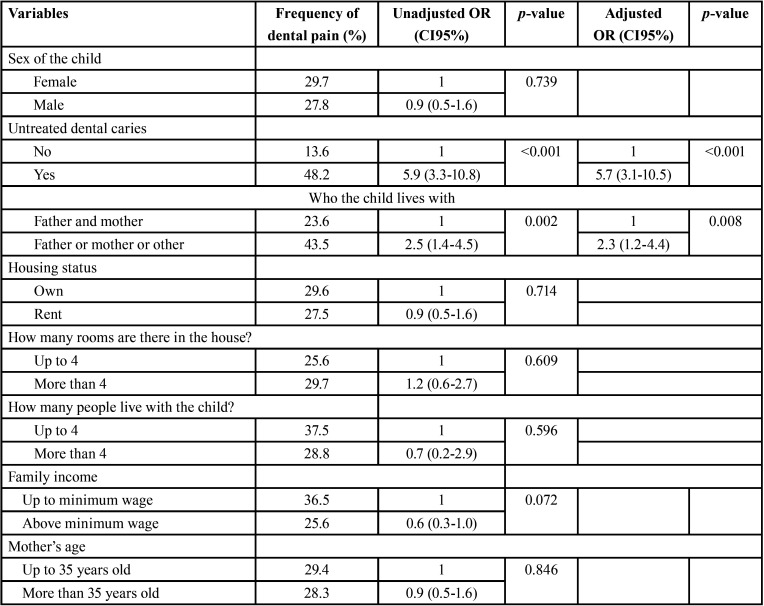
Factors associated with dental pain among 5-year-old children, Brazil, 2019.

**Table 1 cont.**
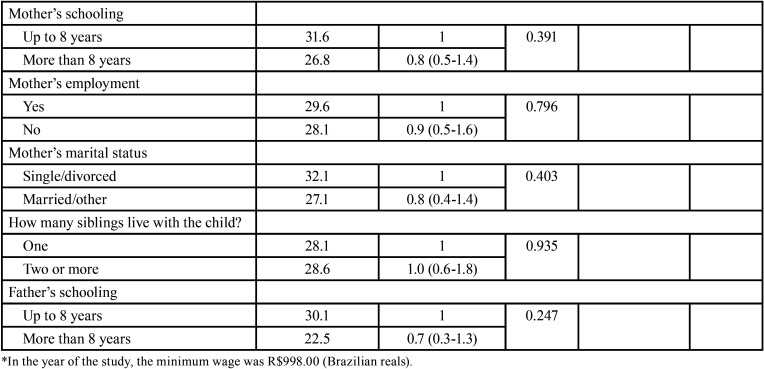
Factors associated with dental pain among 5-year-old children, Brazil, 2019.

## Discussion

According to parents’ reports, the relative prevalence of tooth pain among their children was 28.7% in this study of the Northeast of Brazil in small cities population sizes. Previous studies in Brazil observed similar (1,4) or lower prevalences (3,7,14,16). The difference between this study and previous studies is due to city population size, cultural and organizational aspects, differences in the access to dental services in the regions where the studies were carried out, the sampling methods and the different methods used for data collection. The prevalence of dental caries was 48.2% in this study.

In this study, an association was found between toothache and the presence of untreated dental caries in 5-year-old children. Similar results were found in other studies with different city size population (1,3,4,6,7,14,20,21). The WHO criteria for dental caries recommend that teeth with cavities must be classified as decayed. The presence of deep cavities is associated with dentin exposure or, in the most severe cases, with pulp inflammation.

Children who lived with only one parent or with another person from the family were 2.3 times more likely to have toothache compared to those who lived with both their father and mother. Another study has shown that living with only one parent (either the mother or the father) is a risk factor for the presence of dental caries (22).

The social support that a child receives is associated with the behaviors of their caregivers, including their oral care. Caregivers play an important role in shaping habits and home routines (23,24). Similarly, a previous study showed that the experience of dental caries was more common among children who lived with their grandparents, possibly due to the parenting style and higher prevalence of sugary foods. In that study, most of the parents were single and had less control over the snacking and teeth brushing practices of their children (25). However, there is no consensus in the literature on this issue. A study in China observed that the social support from caregivers was not associated with the consumption of sugary snacks or brushing frequency (10).

There was no association between toothache and sociodemographic factors in this study, consistent with the literature (3,14,26). However, previous studies have reported an association between toothache and low levels of parental education and family income; children of parents with low levels of education and income tend to experience more dental caries and, consequently, more dental pain (26).

Studies on the association between toothache and sex are inconclusive. In the present study, the sex of the child did not remain in the final model, consistent with other studies (3,14).

Dental pain is a relatively common condition that affects quality of life (1,3,11). From this perspective, analyzing this condition and the factors associated with it is of great relevance for obtaining data and planning interventions.

In view of the results regarding the prevalence of toothache and untreated dental caries, there is a need to establish public oral health policies aimed at 5-year-old children and their families, especially policies that facilitate intersectoral actions. The existing actions and activities regarding primary care in Brazil (17) need to be improved by expanding access to oral health services and preventive measures to provide comprehensive health care for children and their families.

Based on the present study, toothache presents itself as a challenge for access to dental services and organization in small municipalities in the interior of northeastern Brazil, as observed in larger population studies (1,3,4,7,27).

This study has limitations. This is a cross-sectional study, and thus, it was not possible to establish causal relationships between the variables. Parents or guardians answered a single question about toothache, and there was no further information about toothache episodes during the children’s life. Many studies have used this same methodology; however, the questions refer to the participants’ memory, which can generate bias. On the other hand, the study was preceded by a pilot study; the questionnaire has questions that have been used in previous studies; and the examiners were trained with a dental caries index that was developed by the WHO and is used worldwide. Longitudinal studies would be useful for studying toothache in children under 5 years old.

It was observed that in Northeast of Brazil countryside cities with small population sizes the prevalence of dental pain in preschool children was high, and this condition is associated with untreated dental caries and living with only one parent or other family members. Sociodemographic factors were not associated with dental pain.
